# Ab Initio Cluster Approach for High Harmonic Generation in Liquids

**DOI:** 10.1021/acs.jctc.2c00235

**Published:** 2022-06-14

**Authors:** Ofer Neufeld, Zahra Nourbakhsh, Nicolas Tancogne-Dejean, Angel Rubio

**Affiliations:** † Max Planck Institute for the Structure and Dynamics of Matter and Center for Free-Electron Laser Science, Hamburg 22761, Germany; ‡ Center for Computational Quantum Physics (CCQ), The Flatiron Institute, New York, New York 10010, United States

## Abstract

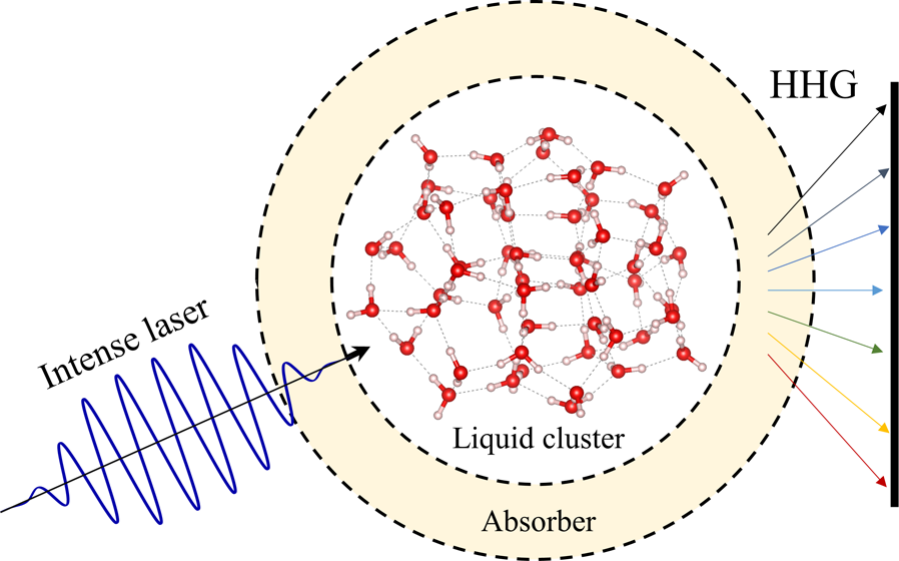

High harmonic generation (HHG) takes place in all phases of matter. In gaseous atomic and molecular media, it has been extensively studied and is very well understood. In solids, research is ongoing, but a consensus is forming for the dominant microscopic HHG mechanisms. In liquids, on the other hand, no established theory yet exists, and approaches developed for gases and solids are generally inapplicable, hindering our current understanding. We develop here a powerful and reliable ab initio cluster-based approach for describing the nonlinear interactions between isotropic bulk liquids and intense laser pulses. The scheme is based on time-dependent density functional theory and utilizes several approximations that make it feasible yet accurate in realistic systems. We demonstrate our approach with HHG calculations in water, ammonia, and methane liquids and compare the characteristic response of polar and nonpolar liquids. We identify unique features in the HHG spectra of liquid methane that could be utilized for ultrafast spectroscopy of its chemical and physical properties, including a structural minimum at 15–17 eV that is associated solely with the liquid phase. Our results pave the way to accessible calculations of HHG in liquids and illustrate the unique nonlinear nature of liquid systems.

## 1 Introduction

High harmonic generation (HHG) is an extremely nonlinear optical process that occurs when intense laser fields are irradiated onto material media. Interactions between electrons in the medium and the incident laser result in an up-conversion of photons, emitting a spectrally wide frequency comb that reaches up to XUV energies.^1^ HHG has been experimentally demonstrated in all phases of matter, namely, in gases, solids, and liquids. In the gas phase, where it was first discovered,^2,3^ HHG has been extensively researched for several decades and is very well understood. Here, it is commonly described by a semiclassical^4−6^ (or quantum^7,8^) three-step model that intuitively describes the process in three sequential steps: (i) ionization of an electron due to laser-induced suppression of the binding coulomb potential, (ii) acceleration of the liberated electron in the continuum whereby it gains kinetic energy, and (iii) a recombination of the liberated electron with its parent ion (or ions in molecules) that results in the emission of high-energy photons. This model is routinely used to explain experimental results and to develop new spectroscopy and interferometry approaches. Notably, the three-step model relies on the fact that the atoms or molecules in the gas are isolated in real space.

In recent years, it was shown that solids are also a prominent source of high harmonics with some possible advantages over their gas-driven counterparts.^9,10^ The mechanism for HHG in solids differs from that in gases and mainly relies on the interference from two types of emissions: (i) emission due to intraband motion of electrons within the nonparabolic band structure and (ii) interband emission due to electron–hole recombination, which is analogous to the gas-phase three-step model except that electrons accelerate along the bands in *k*-space.^11−19^ Notably, the theory that describes these mechanisms relies on long-range translational symmetries of the solid (i.e., a band structure picture). It is important to point out that a fundamental understanding of the HHG process in both gases and solids is the driving force behind technologies and applications based on high harmonics, e.g., attosecond pulses,^1,20,21^ novel ultrafast spectroscopies,^22−29^ and imaging techniques.^30−34^ From a practical standpoint, developing new ultrafast (and potentially attosecond) spectroscopies based on HHG is highly appealing since the spectra are usually extremely sensitive to any internal structure in the nonlinear media (e.g., its symmetry,^35−38^ topology,^29,39−43^ chirality,^44−48^ etc.) due to the highly nonlinear nature. The prospects of transferring some of the ideas and methods implemented in gases and solids to the liquid phase are exciting since most biochemical processes occur in liquid or hydrated phases.

Contrary to gases and solids, liquid HHG measurements are quite scarce. HHG was observed in liquid microdroplets^49^ and surfaces^50^ and in the perturbative regime from bulk liquids.^51^ More recently, XUV high harmonics were measured and characterized from bulk liquids.^52^ Here, there are many fundamental open questions. For instance, the HHG cutoff scaling law is still under debate,^52^ the dominant generation mechanisms have yet to be uncovered, and it is not yet clear how the process depends on the physical and chemical properties of the liquid. A few theoretical works attempted to answer some of these questions,^53−55^ but they relied on a one-dimensional (1D) toy model of a linear chain of atoms with introduced disorder rather than a realistic liquid. Such models fail to capture many of the intricate properties of liquids, especially those relying on the symmetry of the medium and its short-range structure beyond just intermolecular distances. Indeed, the main challenges for a description of liquids are that one usually needs large molecular ensembles to correctly capture their short-range coordination and isotropic nature, while many hybridized molecular states are chemically and optically active. Other effects such as hydrogen bond dynamics are also notoriously difficult to simulate.^56−58^ At the same time, mechanisms and analytic approaches developed for gaseous and solid media are inapplicable to liquids because they lack long-range correlations and are dense infinite systems. Nevertheless, a proper and feasible description of strong light–matter interactions in liquids is necessary to answer all of the fundamental questions above, and the lack of such an approach is one of the main reasons that liquid HHG has been poorly understood thus far.

Here, we develop an ab initio approach for strong light–matter interactions in realistic liquids interacting with arbitrarily polarized laser pulses. The scheme is based on time-dependent density functional theory (TDDFT) for large molecular clusters, although it can also be implemented with other ab initio techniques. To make calculations feasible, we utilize several approximations for the dynamics: (i) dynamical electron–electron correlations are frozen in time, (ii) contributions of surface-localized states to the nonlinear response are suppressed, (iii) contributions of deep-lying states to the nonlinear response are neglected, and (iv) the cluster response is orientation averaged to mimic an isotropic system. These approximations are tested explicitly and also by comparing them to experimental results.^59^ The model is then employed for HHG calculations in liquid water, ammonia, and methane. We compare the characteristic response of polar and nonpolar liquids and find that nonpolar liquids lead to much sharper harmonic peaks with suppressed interference effects. We show that the HHG spectra from liquid methane contain interesting features that could be used for ultrafast spectroscopy, including a structural minimum at 15–17 eV and a well-like shape in the perturbative region.

The paper is organized as follows. In Section 2, we introduce our approach and the logic behind it. In Section 3.1, we analyze HHG in liquid water in various laser conditions. Section 3.2 addresses the main differences between HHG from polar and nonpolar liquids. Finally, Section 4 summarizes our results and presents an outlook.

## 2 Method Formulation

**Figure 1:**
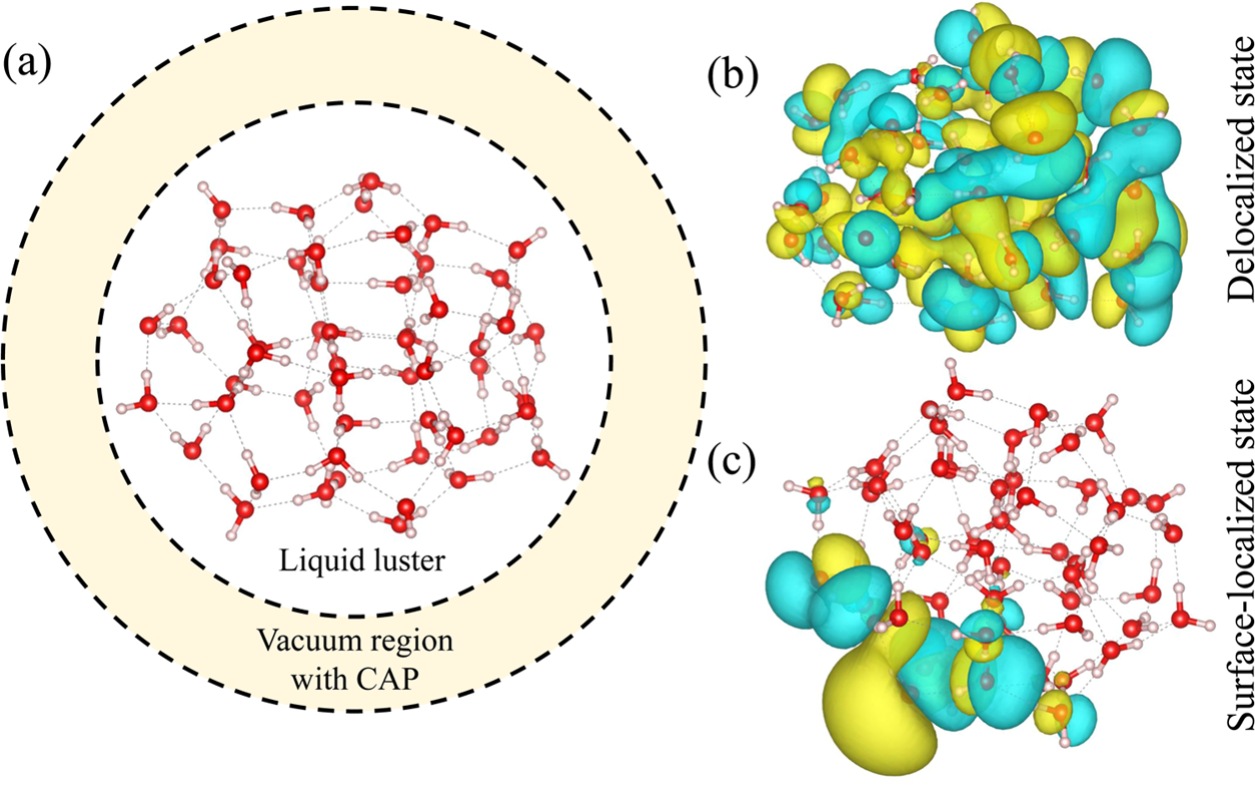
Conceptual illustration of the proposed cluster approach to study HHG in liquids. (a) Approximately spherical cluster with a geometry and density that corresponds to a liquid phase is embedded in a real-space grid with a spherical boundary. The cluster is encapsulated by an absorbing layer that passivates the nonlinear response associated with the surface to mimic a bulk liquid. This illustration depicts a liquid water cluster with 54 H_2_O molecules obtained from ref (61). (b) Exemplary 151st Kohn–Sham (KS) state that is delocalized and is part of several bands of delocalized states. (c) Same as (b), but for the 216th KS state that is highly surface-localized and is part of a localized top-most band of states.

We begin with a formal description of our approach. The liquid is described with relatively large molecular clusters of 40–60 molecules. The geometries of the clusters can be readily obtained as minimal energy configurations^60,61^ or from molecular dynamics simulations^62−64^ (see illustration in Figure 1a for minimal energy configuration of a water cluster). The ground state of each cluster is obtained using real-space grid-based density functional theory (DFT) calculations with the octopus code.^65−68^ The real-space formulation allows us to employ a minimal spherical box shape that has additional vacuum spacing, where the vacuum layer size is defined as the distance between the outermost atom in the cluster and the box wall. In this paper, we consider Perdew–Burke–Ernzerhof (PBE) exchange-correlation (XC)^69^ with an added van der Waals correction,^70^ and core states are replaced by norm-conserving pseudopotentials.^71^ Notably, semilocal XC functionals are known to lead to some deficiencies, which can be corrected for instance by implementing improved meta-generalized gradient approximations (meta-GGA) or hybrid functionals.^72−79^ These are much more computationally intensive, which is why in this first study we employ semilocal XC approximations (but the method can be implemented with any functional of choice). We neglect here the spin degree of freedom for simplicity. Additional technical details are delegated to the Supporting Information (SI).

Upon obtaining the Kohn–Sham (KS) orbitals that comprise the ground state electron density, we analyze their structure to identify any surface-localized bands as opposed to delocalized states (see illustration in Figure 1b,c). This step is crucial since the large surface-to-volume ratio of clusters often leads to significant localization even for closed-shell molecules. However, our goal is to describe the response of bulk liquids. Accordingly, surface-localized states need to be cataloged such that their response in the time-dependent calculations can be removed. In that respect, we artificially divide the ground state electron density into “surface-localized” and “bulk” contributions
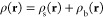
1where ρ_s_(**r**) denotes the density of surface-localized states that is comprised by summing the ground state densities of occupied surface-localized KS states: ρ_s_(**r**) = ∑_*j*∈*s*_|⟨**r**|φ_*j*_^KS^⟩|^2^, with “s” representing the set of surface-localized states and |φ_*j*_^KS^⟩ being the *j*th ground state KS orbital. The “bulk” contribution to the density, ρ_b_(**r**), is given by subtracting ρ_s_(**r**) from the total ground state density, ρ(**r**), with eq 1.

The next question is how to determine which states are surface-localized. For instance, one option is to integrate the density of each KS state over a region localized along the cluster’s surface to provide the percent of surface-localized charge, with which a criterion can be established for determining if a state is localized or not. Alternatively, one may calculate the expectation value ⟨φ_*j*_^KS^|**r**^2^|φ_*j*_^KS^⟩ for each KS state, which estimates the spatial extent of a given KS orbital to produce a similar quantitative criterion. In any case, we note that these choices are not unique, and other conditions could be employed. In this work and as an initial intuitive approach, we denote the last band of occupied orbitals in polar liquids as surface-localized. The terminology “band” refers to a set of KS orbitals that have quasi-continuous KS eigenvalues and are gapped from neighboring such bands. Even though this last occupied band of orbitals was found to be predominantly surface-localized in polar liquids, it also includes a small number of orbitals that are delocalized. Similarly, inner bands of orbitals include a small number of states that are surface-localized. Still, we have found that our results are weakly dependent on the inclusion/exclusion of a small number of these orbitals due to the system size and their localized nature (see the SI for further discussion), such that this is an effective approach to freeze the dominant surface response in the examined systems. It is also noteworthy that surface localization is the result of bonding between molecules, e.g., through hydrogen bonds, van der Waals interactions, or other sources. Consequently, weakly bonded liquids (e.g., liquid helium) do not require this procedure.

In the next step, we wish to describe the interaction of the liquid with an incident laser pulse. This is accomplished within TDDFT, where the KS orbitals are propagated with the following coupled equations of motion (we use atomic units throughout)
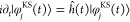
2where |φ_*j*_^KS^(*t*)⟩ is the *j*th time-dependent KS state and *ĥ*(*t*) is the one-body Hamiltonian
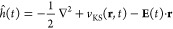
3and where *v*_KS_(**r**,*t*) is the time-dependent KS potential that is given in the adiabatic approximation by
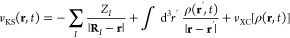
4Here, *Z*_*I*_ is the charge of the *I*th nuclei in the cluster and **R**_*I*_ is its coordinate, *v*_XC_ is the XC potential that is a functional of ρ(**r**,*t*) = ∑_*j*_|⟨**r**|φ_*j*_^KS^(*t*)⟩|^2^, the time-dependent electron density. The motion of the nuclei is neglected, which is justified for interactions with ultrashort laser pulses (even in longer pulses, effects are expected to be small^80−82^). Note that the bare Coulomb interactions of electrons with the nuclei in eq 4 are replaced by pseudopotentials in calculations to reduce computational costs. **E**(*t*) in eq 3 is the electric field vector of a laser pulse with an arbitrary polarization and carrier frequency. We use the dipole approximation and neglect the spatial dependence of the electric field, which is justified for laser wavelengths that are much larger than the cluster sizes. Accordingly, we also neglect interactions with the magnetic field components of the laser and any other relativistic terms such as spin–orbit coupling (these can be added in a straightforward manner). We use the length gauge for describing the light–matter interaction term, but equivalent forms can also be utilized. Finally, we note that the initial KS orbitals are taken as their ground state forms.

Before solving these equations, we must address several points: (i) Remove any surface and finite-size effects from the response to capture only bulk contributions. (ii) Recall that unlike the bulk liquid, the cluster is not perfectly isotropic (this is true even for large clusters). We address point (i) by freezing the localized surface states to their initial form, i.e., |φ_s_^KS^(*t*)⟩ = |φ_s_^KS^(*t* = 0)⟩ for any “s” that corresponds to surface-localized states. This guarantees that inner molecules in the cluster feel a mean-field potential that is still affected by surrounding electrons (because the outer-shell molecules do not get ionized and still contribute to the Hartree and XC terms). Furthermore, the surface states themselves do not contribute to the response of the liquid because they are kept static. Practically, this means that the total electron density, ρ(**r**,*t*), is divided into a dynamical piece that is allowed to evolve, ρ_dyn_(**r**,*t*), and a frozen piece that corresponds to the static charge density, ρ_s_(**r**). We also suppress any additional surface response by adding a complex absorbing potential (CAP) to the vacuum region (see illustration in Figure 1a and details in the SI).^83^ This means that a non-Hermitian term, *v*_CAP_(**r**), is added to the total KS potential in eq 3 during temporal evolution. Importantly, this CAP is placed close to the cluster’s surface to absorb outgoing electrons and prevent them from recombining with the cluster (thus leading to an atomic-like HHG response). Point (ii) is addressed by performing an orientation averaging of the cluster’s response through trapezoidal weights (see the SI for details), i.e., one must perform several calculations with the laser polarization axis rotated in three-dimensional space. These procedures are motivated by the assumption that the liquid response should correspond to that of the inner caged molecules in the cluster since those feel the correct bonding with neighboring molecules and have the proper short-range symmetry and coordination. The orientation averaging is meant to mimic the isotropic response of a much larger liquid volume that is inaccessible in calculations such that the laser “sees” many intermolecular configurations. This is a critical step in our procedure since without it the nonlinear response will not be isotropic (it also increases the numerical costs of performing calculations in liquids). Practically, this should be analogous to experiments, where the nonlinear response arises from a large liquid volume and is averaged over many laser shots. We wish to clarify that to some extent these approximations are performed here ad hoc, even though they are motivated by sound physical intuition. Their purpose is to reduce the immense numerical costs of performing time dependent calculations that employ huge supercells and molecular dynamics simulations. As we will argue below by comparison to experiments,^59^ the approach turns out to perform very well in our examined conditions (although it is possible that it would fail in other regimes or more complex systems where long-range interactions become important). We also note that the particular choice of which surface orbitals are frozen can affect the results and should be explored in future developments.

At this point, we note that even after having performed the approximations above, solving the set of coupled TDDFT KS equations for the cluster is a challenging task. For instance, for a modest cluster size of 50 molecules where each molecule contributes just four active states, there are 200 active orbitals that need to be propagated in tandem, self-consistently, and on large real-space grids. This needs to be performed consecutively for many laser orientations (e.g., 14 orientations for liquid water with linearly polarized light, but the number depends on the molecule and laser conditions) and for a reasonably long simulation time (to obtain spectrally resolved harmonics). To make calculations more accessible, we employ additional approximations. First, we freeze the KS potential to its ground state initial form, i.e., *v*_KS_(**r**,*t*) = *v*_KS_(**r**,*t* = 0), which fully uncouples the equations of motion for the KS orbitals. This approximation is valid only for relatively moderate laser powers where ρ(**r**,*t*) does not change drastically. It is the equivalent of the noninteracting electron approximation that has seen great success in both gas and solid HHG.^1,18,19,21,84^ We test this approximation and make sure that it is valid in the SI. Second, we freeze the response of any deeper-lying states that contribute negligibly to the optical response. This is analogous to the single-active-electron approximation that is standardly used in gas-phase HHG^1^ but where only the lowest-energy band of states is frozen (it is also analogous to limited-band models in solid HHG^19,21^). Altogether, we are left with uncoupled equations of motion for the remaining orbitals (those that are not deep-lying nor surface-localized), which constitutes a significant reduction in the problem size.

Upon propagating the KS orbitals (see the SI for numerical details), we obtain ρ(**r**,*t*), from which the induced microscopic polarization is given as
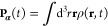
5where α denotes the solid-angle orientation of the cluster with respect to the laser. Following orientation averaging, the induced polarization of the isotropic liquid is given as
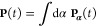
6The dipole acceleration, **a**(*t*), is found directly by the second temporal derivative of **P**(*t*). The harmonic spectrum is given by the Fourier transform of **a**(*t*): *I*(Ω) = |∫d*t* **a**(*t*) e^–iΩ*t*^|^2^.

It is helpful to briefly summarize the numerical parameters of the approach to be converged. First, there are the standard parameters for the DFT calculations of the ground state, e.g., spacing and grid dimensions. Second, there are numerical parameters of the propagation scheme, including time-steps, the vacuum spacing, and parameters of the CAP. Finally, there are the conceptual details of the model: the cluster size and the angular orientation grid density. It is noteworthy that the degree to which a spectrum is considered “converged” varies depending on the physical quantity being studied. For instance, the HHG cutoff tends to converge much faster than the individual harmonic powers. Also, the symmetry of the specific liquid can greatly affect the speed of convergence with respect to cluster size and orientations. At the very least, the cluster size should be large enough to contain several inner caged molecules with the correct coordination. Convergence data is presented in the SI.

As a final note on the feasibility and accessibility of the approach, we highlight the order of magnitude of the required resources—we obtain single-converged HHG spectra for a linearly polarized laser (at an 800 nm wavelength with eight optical cycle long pulses (21.3 fs)) from clusters with ∼50 molecules (∼100 active KS states) and with ∼15 orientations in ∼15,000 CPU hours. Parallelized over 256 CPUs, this is ∼2 days per spectra. These figures are comparable in magnitude to those required from TDDFT calculations for solid HHG (depending on the system).

## 3 Results and Discussion

### 3.1 HHG in Liquid Water

Having outlined our approach, we now utilize it to perform HHG calculations in liquids. For simplicity, we explore monochromatic linearly polarized laser pulses of the form
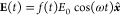
7where ω is the fundamental frequency, *E*_0_ is the field amplitude, and *f*(*t*) is a trapezoidal envelope function with two-cycle long rise and drop sections and a four-cycle long flat top section.

**Figure 2:**
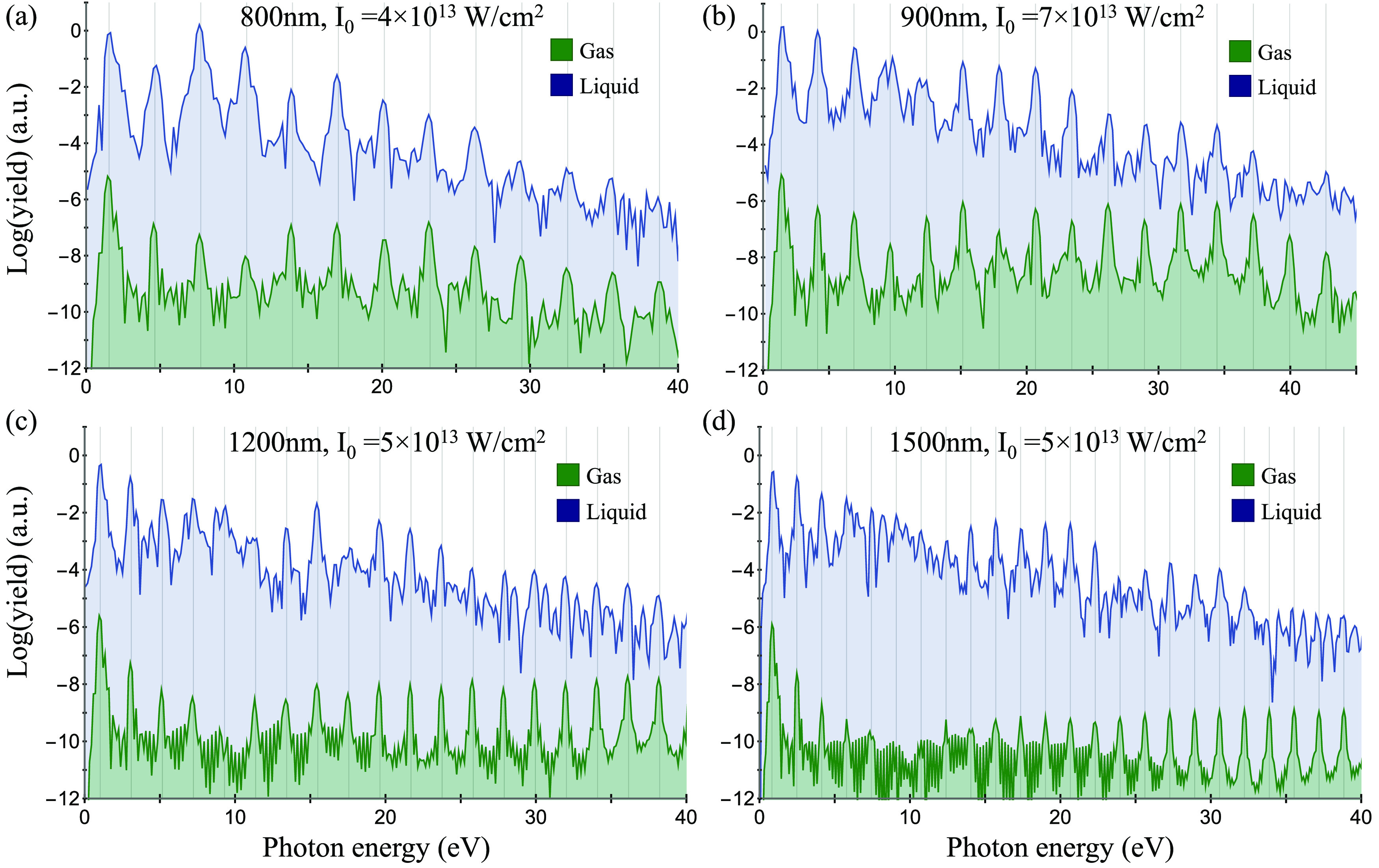
HHG spectra calculated with the cluster approach for liquid water (blue) at various laser conditions (a–d). Green spectra represent calculations for the single-molecule gas-phase case in similar settings (these have been artificially reduced in power to enhance visibility). Gray lines denote positions of odd harmonics.

We begin by analyzing HHG in liquid water. Cluster geometries were obtained from ref (61) as minimal energy configuration of the AMOEBA force-field approach^85^ (see Figure 1a). In water, we find that the highest-energy hybridized band of orbitals is largely surface-localized (see illustration in Figure 1c). The deepest band of orbitals contributes negligibly to the HHG response (see the SI). Thus, there are two active bands comprised of *N* orbitals each (*N* being the number of molecules in the cluster). Figure 2 presents exemplary HHG spectra obtained at various laser wavelengths and powers. It is immediately apparent that the spectra in Figure 2 contain only odd harmonics (indicated by dashed gray lines in Figure 2). We further note that all harmonics have only *x*-polarized components. These fundamental symmetry constraints^38,86^ indicate that the nonlinear optical response of the cluster is indeed isotropic, as required from a bulk liquid. It also suggests that the surface response is correctly suppressed, because it would result in nonisotropicity. We highlight that the procedure for freezing the dynamics in surface-localized states is essential, as the HHG spectra are quite sensitive to the inclusion of these orbitals. This is expected given that they have a much lower ionization potential and are less strongly bounded. Thus, without employing this approach, the cluster is expected to lead to HHG spectra that is associated with a surface rather than a bulk response.

Figure 2a,b also compares these results to HHG calculations from a single gas-phase isolated water molecule (calculated on a similar level of theory, see the SI for details). The HHG cutoff from the single-molecule case considerably deviates from the cutoff in the liquid, hinting toward the different mechanisms active in each system (dilute gases or liquids). Notably, there is some emission noise beyond the cutoff of the liquid that corresponds to energy ranges where harmonics are still emitted from the gas (e.g., see noisy emission beyond 35 eV in Figure 2b). This noise can be interpreted as HHG contributions from electrons that are ionized from the cluster but are adequately absorbed by the CAP. Overall, we conclude that the approach indeed manages to suppress surface contributions and mimic the isotropicity of a bulk liquid. Importantly, these results hold for all examined laser parameters (wavelengths of 800–1500 nm and intensities of 3–8 × 10^13^ W/cm^2^) and liquids, supporting the generality of the technique. In the SI, we explore the specific contributions of different bands of KS orbitals to the full HHG response and show that the top-most two bands are both essential to obtain the spectra—contributions from both of these bands are on the same order of magnitude and interfere either constructively or destructively, depending on the harmonic order.

At this point, we highlight that the numerical technique developed here was recently used to explore the cutoff scaling of liquid HHG with respect to the wavelength and to derive an intuitive picture of the HHG mechanism in liquids.^59^ Reference (59) has demonstrated that the cluster approach successfully reconstructs the experimentally measured wavelength-independent cutoff (contrary to the standard behavior in gases and solids^1,21^). Moreover, in ref (59), we compared our results to ab initio calculations based on large supercells within a molecular dynamics approach (that are much more expensive), and the results qualitatively agree with results from the cluster approach. This further establishes the validity of the model and its utilization for exploring fundamental phenomena in liquids.

### 3.2 HHG from Polar and Nonpolar Liquids

**Figure 3:**
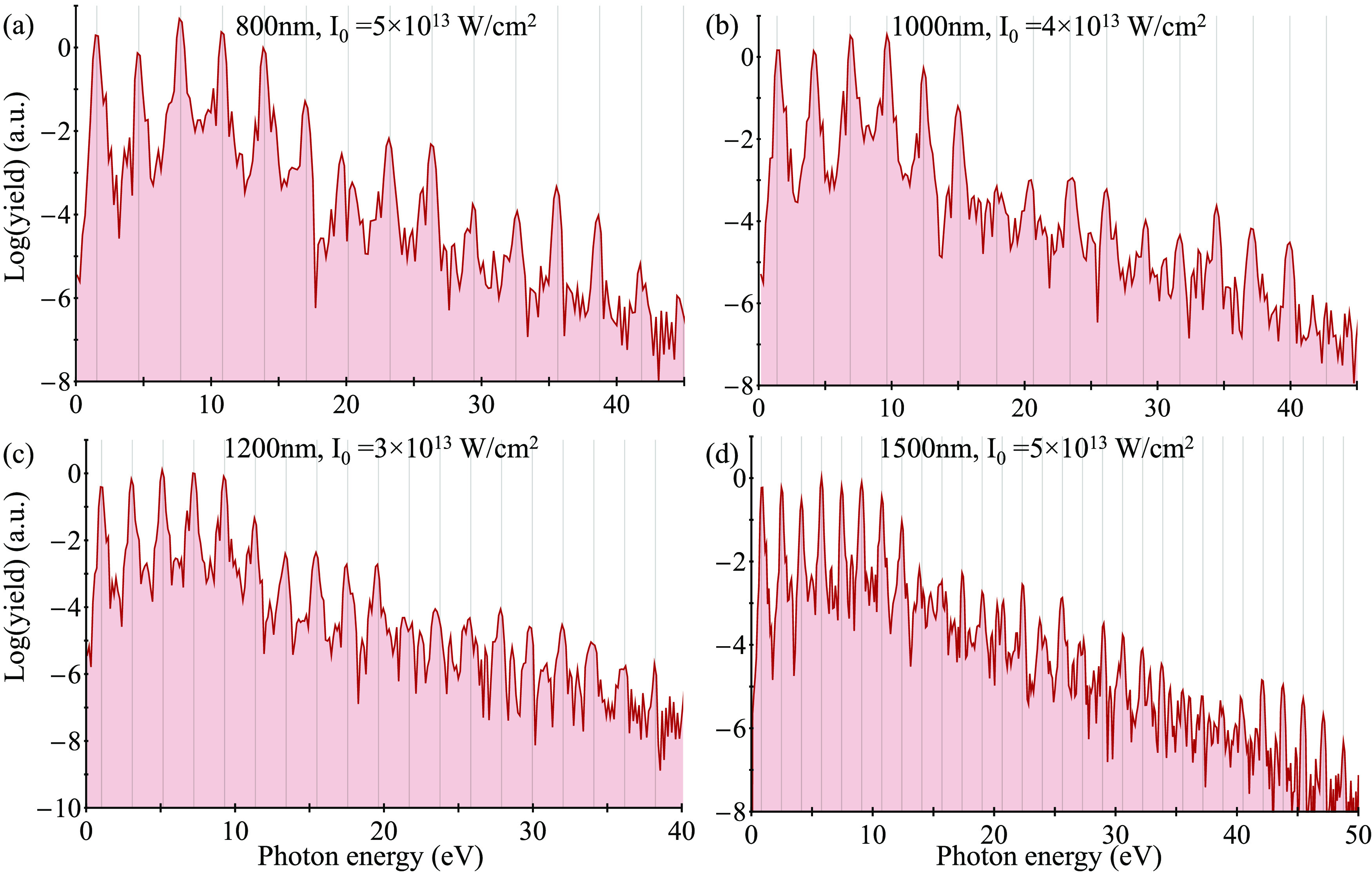
HHG spectra calculated with the cluster approach for liquid ammonia at various laser conditions (a–d). Gray lines denote positions of odd harmonics.

**Figure 4:**
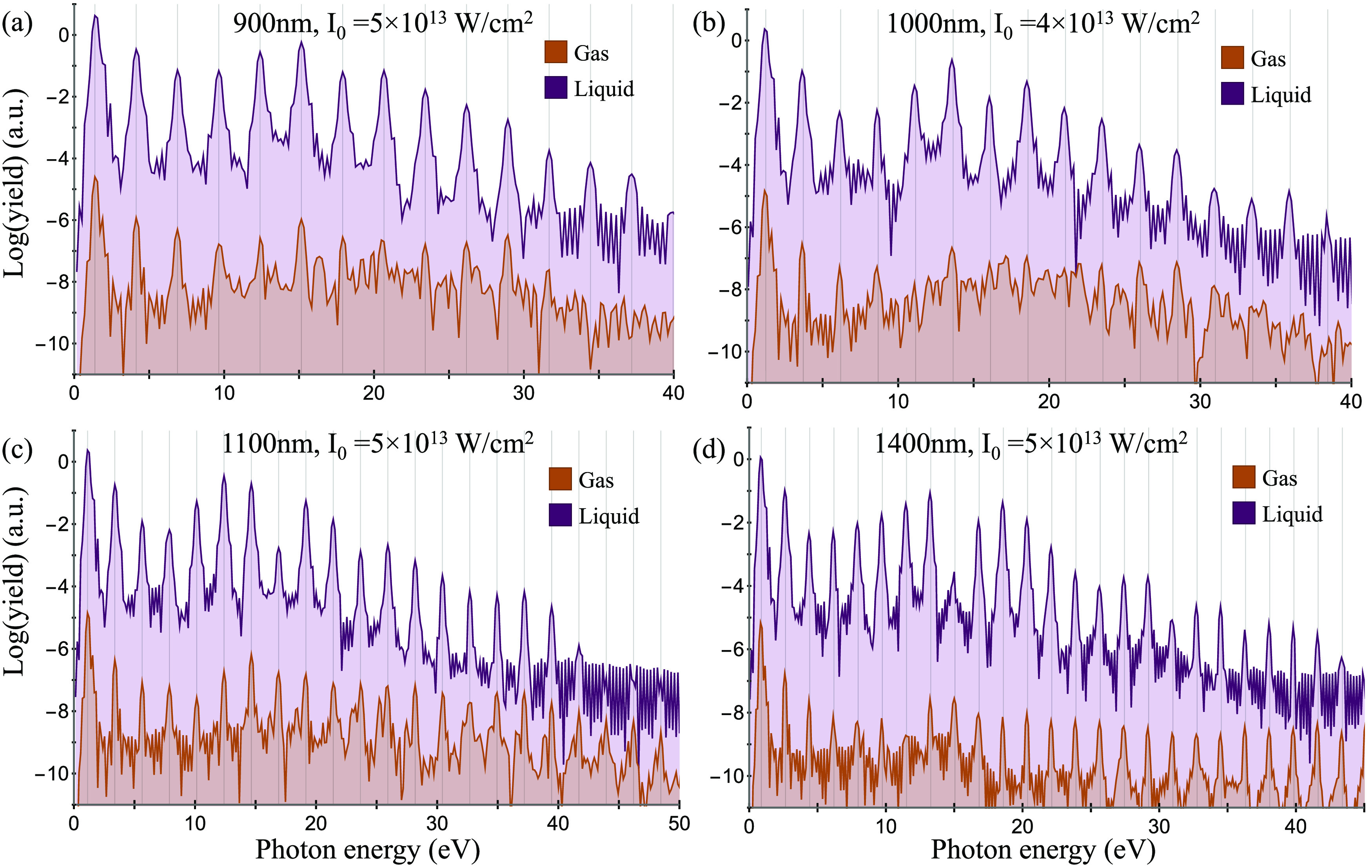
HHG spectra calculated with the cluster approach for liquid methane (purple) at various laser conditions (a–d). Orange spectra represent calculations for the single-molecule gas-phase case in similar settings (these have been artificially reduced in power to enhance visibility). Gray lines denote positions of odd harmonics.

We next explore HHG from two additional liquids: ammonia and methane. Liquid methane cluster geometries are obtained from ref (60) as minimal energy configurations based on ab initio obtained potentials,^87^ while liquid ammonia geometries are taken from ref (63) utilizing a molecular dynamics approach. We repeat the calculations performed in the previous part for both of these liquids and scan various laser wavelengths and powers. Figures 3 and 4 present results of similar nature to those seen in water: only *x*-polarized odd harmonics are emitted, indicating that the response is isotropic. We also note that a converged isotropic response for these molecules is obtained for less orientations compared to the case of H_2_O as a result of their higher symmetry (see the SI for details).

It is worthwhile to examine the characteristic differences between the nonlinear response of these various liquids. Most notably, both ammonia and water exhibit strong intermolecular bonding that arises from van der Waals interactions and hydrogen bonding, unlike in methane where each molecule is nearly nonbonded to its neighbors. This fundamental difference also means that methane has practically no surface-state localization. Comparing the HHG spectra in Figures 2 and 3 to those in Figure 4, the spectra from methane comprise much sharper distinct harmonic peaks as opposed to water and ammonia. In water, for instance, some harmonics show sideband oscillations (e.g., the harmonic at 30 eV in Figure 2a) or tend to split into subpeaks (e.g., the harmonic at 10 eV in Figure 2b). The same effect occurs in ammonia (see, for example, the harmonics at 20 and 25 eV in Figure 3a). This is likely a result of multiorbital interference in water and ammonia liquids that can originate from intermolecular recombination or scattering. These interference effects can also be understood to arise in the bonded liquids because they exhibit wide energy bands that allow intricate coupled interband and intraband dynamics in *k*-space. For methane, however, this effect does not occur. Its absence suggests that liquid methane exhibits a more dominant single-molecule response. In fact, the “cleanness” of the harmonic peaks in liquid methane is reminiscent of spectra usually obtained from gas-phase calculations of isolated molecules. We attribute this to the nonbonding nature of the nonpolar liquid that suppresses intermolecular interferences (in *k*-space, this can be thought of as arising from the highly energy-resolved bands that have a uniform character, which suppresses interband–intraband interferences). This feature might be useful in future studies for probing hydrogen bonding dynamics during chemical processes.

**Figure 5:**
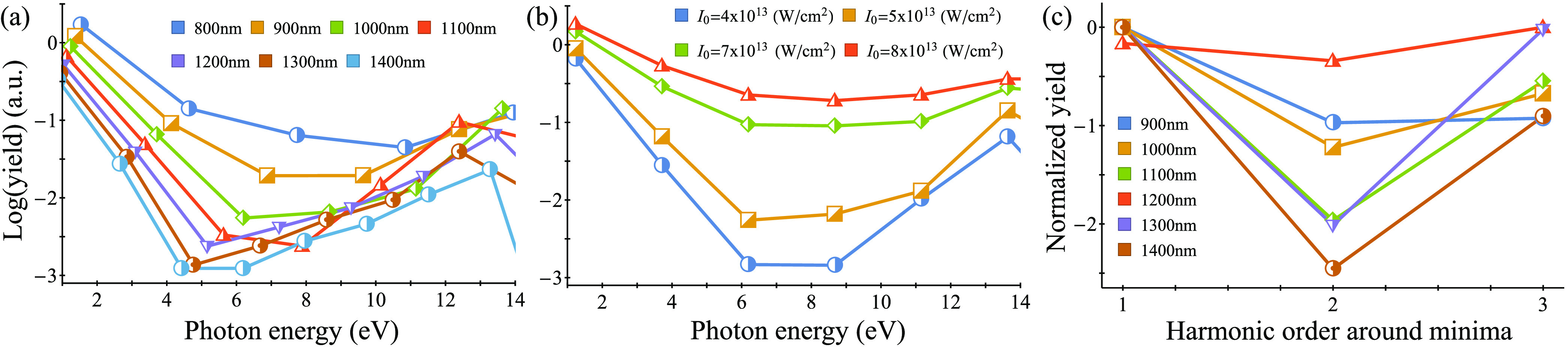
Wavelength and intensity-dependent analysis of well-shaped minima and structural interference in HHG from liquid methane. (a) Integrated harmonic yield per harmonic order in the perturbative region for various laser wavelengths (calculated for *I*_0_ = 5 × 10^13^ W/cm^2^). (b) Same as (a) but for various laser powers (calculated at λ = 1000 nm). (c) Integrated harmonic yield for varying laser wavelengths in the region of a structural interference minimum (15–17 eV), calculated at *I*_0_ = 5 × 10^13^ W/cm^2^. Harmonic yield is presented for three harmonic orders around the minimum in each case (the minimum is shifted to harmonic #2), and the maximal power is normalized to 0.

The exceptionally clean HHG spectra from methane can pose a unique advantage for exploring its structural and electronic properties on ultrafast timescales. This is because it may be more sensitive to small interference effects (whereas in polar liquids, these are more difficult to disentangle). In particular, we note two interesting features that arise in the liquid HHG emission from methane that could be utilized for this purpose:

(i) The harmonic emission at energy ranges up to 14 eV exhibits a very distinct well-like shape with a typical minimum of around 6 eV. That is, the envelope of the harmonic spectra in this energy region has a unique behavior—the yield exponentially drops, reaches a minimum, and then exponentially increases. This is a different behavior than that usually observed in both gases and solids, where the perturbative region shows a simple exponential decay. Figure 5a,b presents the integrated harmonic power from methane liquid in this energy range for various laser parameters—the well shape is observed for a wide regime of laser conditions with wavelengths ranging from 800 to 1500 nm and laser powers of (3–8) × 10^13^ W/cm^2^ (though the specific shape of the well slightly varies with the parameters). We note that a similar effect is observed from methane gas, but it is much weaker compared to the liquid case (see Figure 4). This could indicate that the well originates from the chemical properties of the single CH_4_ molecule but is enhanced when the molecule is dressed by the surrounding environment. In that respect, it could signify features in the HHG response that are specific to intermolecular interactions. This phenomenon is not observed in the other tested liquids (see Figures 2 and 3). The particular shape of the well is reminiscent of that resulting from shape resonances in barrier-well systems, as was demonstrated in (1D) models.^88^ Thus, HHG in liquids could pave the way to novel spectroscopic probing of resonances in the electronic structure.
(ii) The HHG spectra from methane show a clear structural interference minimum at 15–17 eV (see Figures 4 and 5c). The emission at this energy range remains at a stable local minimum even when changing the laser wavelength for a wide range of 900–1500 nm and for laser powers of up to 5 × 10^13^ W/cm^2^. Furthermore, the minimum is not present in gas-phase spectra. Thus, we conclude that it is associated solely with the chemical and physical properties of the liquid system. Notably, the minimum is washed-out at stronger laser powers, possibly indicating that different mechanisms or pathways become dominant. We also note that it is slightly less pronounced when including dynamical correlations in the calculation, though still visible (see the SI). This should be investigated in future work. We emphasize that this is the first prediction of a structural minimum in the HHG spectra of a liquid system, which is equivalent to those seen in gases^89−91^ and solids^92^ and which can potentially be used to probe correlations or other dynamical effects.

## 4 Conclusions and Outlook

To summarize, we have put forward a cluster-based ab initio approach for describing interactions between bulk liquids and arbitrarily polarized intense laser pulses. This technique formally relies on TDDFT but utilizes several approximations that allow affordable and accurate calculations for realistic three-dimensional systems. It has also been validated by agreement with experiments.^59^ We implemented our technique to study HHG from liquid water, ammonia, and methane and investigated the role of the liquid’s chemical properties on the spectra. We concluded that spectra from nonpolar liquids show much sharper harmonic peaks than those from polar liquids that exhibit stronger interference effects. We have also shown that the HHG spectra from liquid methane exhibit some interesting characteristics that may be useful for ultrafast spectroscopy: (i) a local minimum in the perturbative harmonic region that might originate from shape resonances,^88^ which is enhanced in the liquid phase. (ii) An electronic-structure minimum that appears at 15–17 eV, which is the equivalent to those observed in gases^89−91^ and solids.^92^ Both of these features could be utilized to study the various chemical and physical properties of liquids with ultrafast temporal resolution (e.g., dynamical correlations, dynamical polarizability, ion motion, etc.).

Apart from the specific predictions presented here, we believe that our approach might pave the way for feasible and accessible calculations of HHG in liquids, as well as other nonlinear processes including photoionization.^93^ This is a crucial step toward an improved understanding of the active mechanisms for strong-field physics in liquids, which is essential for obtaining novel light sources and ultrafast spectroscopic capabilities.

## Supporting Information Available

Technical details of the methodology, convergence tests, and validation of several of the approximations used (PDF)
