# Supplementary material for: Ab Initio Cluster Approach for High Harmonic Generation in Liquids
Source: J Chem Theory Comput. 2022 Jun 14;18(7):4117–26. doi: 10.1021/acs.jctc.2c00235 (PMC9281394; doi:10.1021/acs.jctc.2c00235)
Supplement: Supplementary file 1 — ct2c00235_si_001.pdf [file ct2c00235_si_001.pdf]

Supplementary information: Ab-initio cluster approach for high harmonic generation in liquids

Ofer Neufeld[1,*], Zahra Nourbakhsh[1], Nicolas Tancogne-Dejean[1], Angel Rubio[1,2,*]

[1]Max Planck Institute for the Structure and Dynamics of Matter and Center for Free-Electron Laser Science, Hamburg, 22761, Germany.
[2]Center for Computational Quantum Physics (CCQ), The Flatiron Institute, New York, NY, 10010, USA.
*Corresponding authors emails: oneufeld@schmidtsciencefellows.org, angel.rubio@mpsd.mpg.de.

This supplementary information (SI) file contains technical details on the numerical calculations and methodology used in the main text, as well as some additional results. Section S1 presents details of the numerics, including convergence testing. Section S2 presents some additional results of liquid HHG and tests for the validity of various approximations used in the main text.

▪ S1: NUMERICAL DETAILS

1. Ground state DFT calculations

All DFT calculations were performed using the octopus code[1–3]. The Kohn-Sham (KS) equations were discretized on a Cartesian grid with a spherical shape of radius 31.6, 34, and 32.8 bohr for the converged clusters of water, ammonia, and methane, respectively, and 45 bohr for the single-molecule calculations. Calculations were performed using the PBE exchange correlation functional in all cases[4]. For the liquid clusters, a van-der-waals correction term was added[5]. For the single molecule cases, a self-interaction correction (SIC) was added in order to capture the long-range asymptotic potential[6]. The frozen core approximation was used for core states, which were treated with norm-conserving pseudopotentials[7]. The KS equations were solved to self-consistency with a tolerance <10^{-7} Hartree, and the grid spacing was converged to Δx=Δy=Δz=0.4 bohr, such that the total energy per electron was converged <10^{-3} Hartree. Cluster geometries were obtained as described in the main text. Single molecule geometries were taken at the experimental configuration.

2. Time-dependent calculations

For time-dependent calculations, the active KS orbitals were propagated with a time step Δt=0.11 a.u. and by adding a complex absorbing potential (CAP) with a width of 11.2 Bohr in the vacuum region[8], where the CAP is placed as close to the cluster as possible while still not absorbing charge in the absence of a laser. The CAP width is similar to that standardly employed in HHG calculations, and was converged against a wider CAP with 15 Bohr width (see Fig. S1). The degree of ionization from the cluster is typically small and below ~2% of the total number of electrons, such that there are only weak dynamical effects on the XC potential. The initial state was taken to be the system's ground-state. The grid size, and time step were tested for convergence. The HHG spectra were obtained as explained in the main text where the dipole acceleration was filtered with a super-gaussian window.

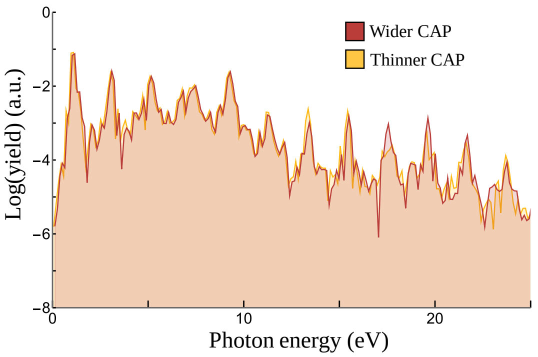

Figure S1. Convergence with respect to CAP width. HHG spectra calculated at 1200nm with a laser power of 4×10^{13} W/cm^2, where the CAP is either 11.2 Bohr wide (yellow), or 16 15 Bohr wide (red). The calculation is performed for a liquid water cluster with 43 molecules.

3. Orientation averaging

Orientation averaging was performed using trapezoidal weights with an angular grid spanned by Euler angles in the *z*-*y*-*z* convention. For the liquids we considered three angular grids with Euler angle spacing of: (1) π/2 for all angles, (2) of π/2 for α and γ and π/4 for β, and (3) π/4 for all angles. After utilizing the symmetries of linearly-polarized light in the dipole approximation, these grids lead to a total of 6, 14, and 58 independent orientations, respectively (which are equivalent to full grids with 75, 125, and 405 orientations, respectively). The HHG spectra in liquid water were converged with grid (2). The HHG spectra of ammonia and liquid methane (which have a higher molecular symmetry than water) were converged with grid (1). For gas-phase calculations we used grid (3).

4. Cluster size convergence

Convergence with respect to cluster size was obtained for a cluster of 54 molecules in the case of water (see Fig. S1(a) showing small disparities for harmonics near 15 and 30eV, but a generally similar spectral structure). For the case of ammonia convergence was obtained for a 56-molecule cluster (see Fig. S1(b) showing small deviations above 30 eV). In liquid methane convergence was obtained for a 40-molecule cluster (see Fig. S1(c) showing small deviations only above 30 eV). In all cases there are small deviations upon increasing cluster size by 10-15%, even though the molecular geometries in each case can be very different (because they were obtained as minimal energy configurations of completely different clusters). This is a good indication for the reliability of the method, and also suggests that the surface contribution is indeed suppressed (because surface reconstruction variations are negligible). Notably, higher energy harmonics are slightly more difficult to converge, and also clusters where the underlying molecule has a lower symmetry converge more slowly (e.g. water). To compensate for this effect and reduce errors even further we use the larger cluster sizes in all calculations.

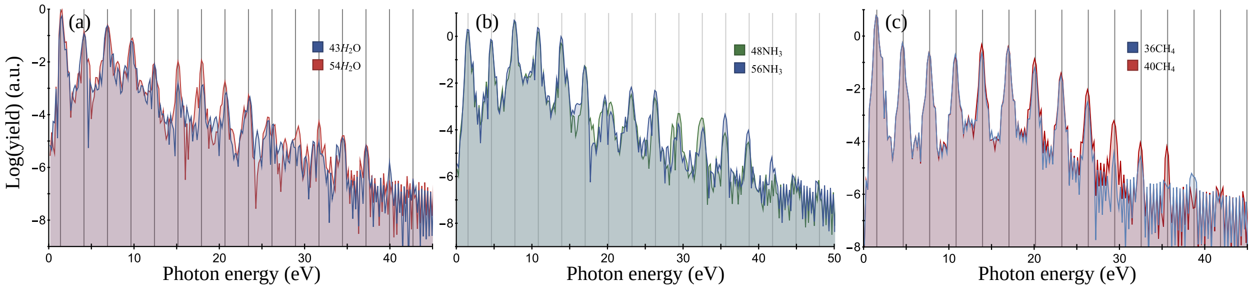

Figure S2. Convergence with respect to cluster size. (a) Liquid water HHG spectra calculated at λ=900nm and a laser power of 4×10^{13} W/cm^2. (b) Liquid ammonia HHG spectra calculated at λ=800nm and a laser power of 5×10^{13} W/cm^2. (c) Liquid methane HHG spectra calculated at λ=800nm and a laser power of 5×10^{13} W/cm^2. Gray lines indicate the position of odd harmonics.

5. Gas-phase calculations

For time-dependent gas phase calculations we utilized exactly the same approach as in the cluster cases. The only differences are that: (1) we performed orientation averaging with a much denser angular grid (see discussion above), and (2) employed a SIC correction rather than a wan-der-waals correction (see discussion above). The same level of theory was used in order to have comparable results between the clusters and the single-molecule calculations, i.e. PBE XC was used throughout and the KS potential was frozen to its initial form.

▪ S2: ADDITIONAL RESULTS

1. Validity of the non-interacting electrons approximation

Throughout the paper we utilized the non-interacting electrons approximation, i.e. the KS potential was frozen to its ground state form. This approximation is standardly used in both gas and solid phases, but has never before been tested for liquids. Here we formally test it for the case of liquid methane (which is simpler due to the lack of surface localization). Figure S2(a) presents HHG spectra from liquid methane obtained at

similar conditions where the KS potential is either kept frozen in time, or allowed to evolve (a standard TDDFT calculation). Some differences indeed emerge between the spectra such as differences in the total harmonic power and small a cutoff value shift. Nonetheless, the envelope structure of the spectra and its characteristic shape is largely unchanged, including for instance the position of the observed interference minima at ~15-17 eV (see main text). We further argue that the source of the disparity between the two approaches is an unphysical renormalization of the KS potential due to spurious ionization from the cluster that is absorbed at the boundaries (as is seen in gas-phase systems[9]). We term this ionization as 'unphysical', since such an effect would not occur in the bulk liquid. To test this, we perform similar calculations at a higher laser power to compensate for the renormalization of the KS potential. Figure S2(b) shows that indeed the full TDDFT calculation at a slightly higher laser power is nearly equivalent to the non-interacting calculation at a lower laser power, indicating that the source of the disparity is a consequence of ionization. In the limit of an infinitely large cluster this effect would not take place; thus, the results overall validate the use of this approximation, at least as a starting point for exploring strong-field physics in liquids.

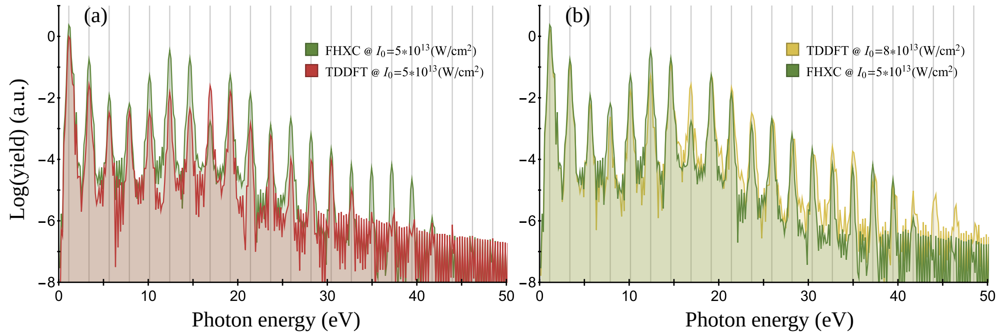

Figure S3. HHG spectra from liquid methane with and without the frozen KS potential approximation. (a) HHG spectra obtained at λ=1100nm and a laser power of 5×10^{13} W/cm^2. (b) The same as in (a), but where the full TDDFT calculations are performed at a laser power of 8×10^{13} W/cm^2 to compensate for the renormalization of the KS potential due to unphysical ionization. Gray lines indicate the position of odd harmonics.

2. Validity of frozen deep-lying states approximation

We demonstrate here the validity of the frozen deep-states approximation used throughout calculations. Figure S3(a) and (b) present HHG spectra from liquid water and ammonia, respectively, where the deepest-lying band of states has been either kept frozen, or allowed to evolve in time (i.e. in the frozen case the lowest N states are frozen where N is the number of molecules in the cluster). Results clearly show that in both cases this approximation is accurate (some disparities arise near 15eV for ammonia, but at an even harmonic order that vanishes after full orientation averaging). Figure S3(c) presents a similar analysis for liquid methane, where it is shown that the response of perturbative (below band gap) harmonics can drastically change if the deepest orbital band of methane is not included in the dynamics. In fact, very large disparities arise for the perturbative harmonics (especially the 5th harmonic) even when only the first 10 states are frozen. Thus, this approximation is not used throughout the paper for methane, only for water and ammonia. The physical source of the disparity in methane remains to be investigated, but could result from polarization of inner states[10]. We emphasize that for higher order harmonics the approximation remains valid just as expected.

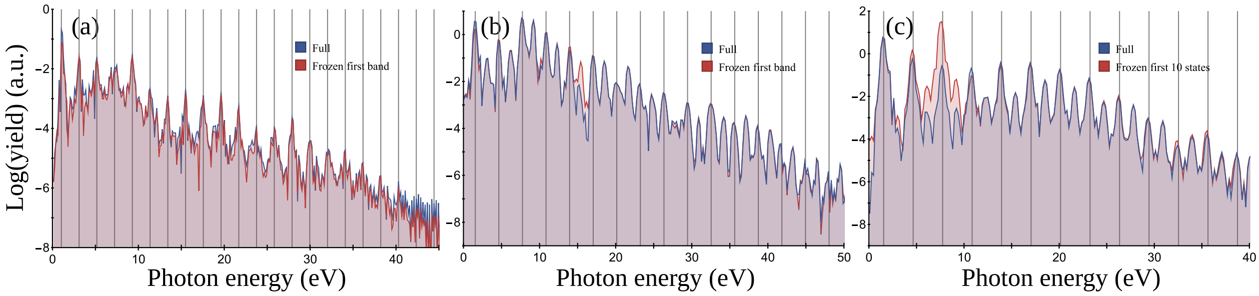

Figure S4. HHG spectra from various liquids with and without the frozen deep-lying states approximation. (a) HHG spectra from water obtained at λ=1200nm and a laser power of 4×10^{13} W/cm^2. (b) HHG spectra from ammonia obtained at λ=800nm and a laser power of 5×10^{13} W/cm^2. (c) HHG spectra from methane obtained at λ=800nm and a laser power of 5×10^{13} W/cm^2. Note that for methane and ammonia the spectra only include a single orientation without averaging, such that even harmonics are also observed. Gray lines indicate the position of odd harmonics.

3. Band-resolved HHG

We explore here the band-resolved contributions to HHG in liquid water for exemplary laser parameters. For the liquid water clusters utilized in HHG calculations, there were two active bands of KS orbitals. These are the topmost band of active orbitals (below the last band of surface-localized states that is frozen), which is denoted as VB1 (valence band 1), and the band just below it that is denoted as VB2. Note that the band notation does not refer to a single orbital, but rather to the full set of orbitals with quasi-continuous KS eigen-energies. Figure S5 shows the HHG spectral contributions from each separate band, and also compared to the full HHG response. A main result is that both of these bands are essential for obtaining the full HHG response, because they have roughly similar order of magnitudes in their nonlinear response. Also, the response from separate bands can interfere both constructively (compare Fig. S5(a) and (b) e.g. at the harmonic at ~20eV), or destructively (e.g. compare Fig. S5(a) and (b) at the harmonic at ~40eV).

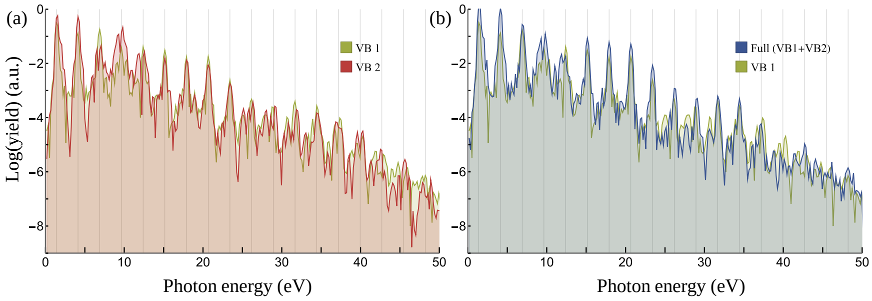

Figure S5. Band-resolved contributions to HHG spectra from liquid water (54 molecule cluster) obtained at λ=900nm and a laser power of 7×10^{13} W/cm^2. (a) HHG spectra from the first and second valence bands of KS orbitals. (b) HHG spectra from the first valence band of orbitals, compared to the full coherent response of the cluster. Gray lines indicate the position of odd harmonics.

4. Surface-state freezing

We further analyze here the choice of which states to consider as frozen in the time-dependent dynamical simulations. In all calculations presented in the main text we chose a simplified approach where the topmost band of orbitals was frozen, while deeper orbitals were propagated. On the other hand, a small number of the inner orbitals still exhibit surface localization by the different measures presented in the main text. Despite this, we have found that inclusion or exclusion of these small number of orbitals changes the HHG response very little, which is presented in Fig. S6 for an exemplary case of HHG in liquid water. Figure S6 compares the HHG spectra obtained in the standard procedure as employed in the main text, to the HHG spectra obtained with freezing an additional 11 surface-localized states within the deeper band of orbitals. The results show that there is a very weak dependence on these states. This probably occurs because of their surface nature which means they are rapidly absorbed in the simulation and do not contribute to the nonlinear response, and also because their number is quite small compared to the total amount of active orbitals. Similarly, we expect that inclusion of a small number of delocalized states from the top-most band would not

change the HHG response substantially, because these states have a very low ionization potential and would be absorbed rapidly by the CAP. Further examination for this procedure should be topic of future studies.

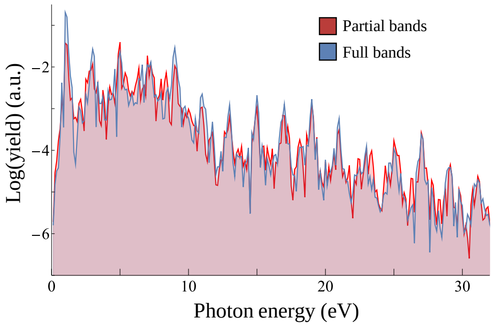

Figure S6. Comparison between HHG spectra calculated with or without freezing additional inner orbitals that are surface-localized, calculated in a 43-molecule cluster of liquid water at 1200nm with a laser power of 4×10^{13} W/cm^2. The notation partial band (red) refers to the case with additional freezing of 11 orbitals that were surface localized, while full band refers to the same approach employed in the main text.

▪ REFERENCES

(1) Castro, A.; Appel, H.; Oliveira, M.; Rozzi, C. A.; Andrade, X.; Lorenzen, F.; Marques, M. A. L.; Gross, E. K. U.; Rubio, A. Octopus: A Tool for the Application of Time-Dependent Density Functional Theory. *Phys. status solidi* **2006**, *243* (11), 2465–2488. https://doi.org/10.1002/pssb.200642067.

(2) Andrade, X.; Strubbe, D.; Giovannini, U. De; Larsen, H.; Oliveira, M. J. T.; Alberdi-rodriguez, J.; Varas, A.; Theophilou, I.; Helbig, N.; Verstraete, M. J.; Stella, L.; Nogueira, F.; Castro, A.; Marques, M. A. L.; Rubio, A. Real-Space Grids and the Octopus Code as Tools for the Development of New Simulation Approaches for Electronic Systems. *Phys. Chem. Chem. Phys.* **2015**, *17*, 31371–31396. https://doi.org/10.1039/C5CP00351B.

(3) Tancogne-Dejean, N.; Oliveira, M. J. T.; Andrade, X.; Appel, H.; Borca, C. H.; Le Breton, G.; Buchholz, F.; Castro, A.; Corni, S.; Correa, A. A.; De Giovannini, U.; Delgado, A.; Eich, F. G.; Flick, J.; Gil, G.; Gomez, A.; Helbig, N.; Hübener, H.; Jestädt, R.; Jornet-Somoza, J.; Larsen, A. H.; Lebedeva, I. V; Lüders, M.; Marques, M. A. L.; Ohlmann, S. T.; Pipolo, S.; Rampp, M.; Rozzi, C. A.; Strubbe, D. A.; Sato, S. A.; Schäfer, C.; Theophilou, I.; Welden, A.; Rubio, A. Octopus, a Computational Framework for Exploring Light-Driven Phenomena and Quantum Dynamics in Extended and Finite Systems. *J. Chem. Phys.* **2020**, *152* (12), 124119. https://doi.org/10.1063/1.5142502.

(4) Perdew, J. P.; Burke, K.; Ernzerhof, M. Generalized Gradient Approximation Made Simple. *Phys. Rev. Lett.* **1996**, *77* (18), 3865. https://doi.org/10.1103/PhysRevLett.77.3865.

(5) Grimme, S.; Antony, J.; Ehrlich, S.; Krieg, H. A Consistent and Accurate Ab Initio Parametrization of Density Functional Dispersion Correction (DFT-D) for the 94 Elements H-Pu. *J. Chem. Phys.* **2010**, *132* (15), 154104. https://doi.org/10.1063/1.3382344.

(6) Legrand, C.; Suraud, E.; Reinhard, P.-G. Comparison of Self-Interaction-Corrections for Metal Clusters. *J. Phys. B At. Mol. Opt. Phys.* **2002**, *35* (4), 1115–1128. https://doi.org/10.1088/0953-4075/35/4/333.

(7) Hartwigsen, C.; Goedecker, S.; Hutter, J. Relativistic Separable Dual-Space Gaussian Pseudopotentials from H to Rn. *Phys. Rev. B* **1998**, *58* (7), 3641–3662. https://doi.org/10.1103/PhysRevB.58.3641.

(8) De Giovannini, U.; Larsen, A. H.; Rubio, A. Modeling Electron Dynamics Coupled to Continuum States in Finite Volumes with Absorbing Boundaries. *Eur. Phys. J. B* **2015**, *88* (3), 56. https://doi.org/10.1140/epjb/e2015-50808-0.

(9) Neufeld, O.; Cohen, O. Probing Ultrafast Electron Correlations in High Harmonic Generation. *Phys. Rev. Res.* **2020**, *2* (3), 033037. https://doi.org/10.1103/PhysRevResearch.2.033037.

(10) Abu-samha, M.; Madsen, L. B. Effect of Multielectron Polarization in the Strong-Field Ionization of the Oriented CO Molecule. *Phys. Rev. A* **2020**, *101* (1), 13433. https://doi.org/10.1103/PhysRevA.101.013433.
